# Gender, socio-economic status and metabolic syndrome in middle-aged and old adults

**DOI:** 10.1186/1471-2458-8-62

**Published:** 2008-02-18

**Authors:** Ana C Santos, Shah Ebrahim, Henrique Barros

**Affiliations:** 1 Department of Hygiene and Epidemiology, University of Porto Medical School, Porto, Portugal; 2 Department of Epidemiology and Population Health, London School of Hygiene & Tropical Medicine, London, UK

## Abstract

**Background:**

Studies that addressed social and economic determinants of cardiovascular diseases, consistently showed an increase prevalence of the individual features of metabolic syndrome in the lower socio-economic strata. Thus, this study aimed to evaluate the association between social class and metabolic syndrome in a sample of urban middle-aged and old Portuguese adults.

**Methods:**

We evaluated 1962 subjects (1207 women and 755 men) aged 40 or more years. Marital status, education, occupation, menarche age and height distribution were used as socioeconomic indicators. Metabolic syndrome was defined according to the ATP III, by the presence of at least three of the following characteristics: waist circumference >102 cm in men and >88 cm in women; triglycerides ≥ 150 mg/dl; HDL cholesterol <40 mg/dl in men and <50 mg/dl in women; blood pressure ≥ 130/85 mm Hg; and fasting glucose ≥ 110 mg/dl. Proportions were compared using the chi square test or *Fisher's *exact test. Odds ratios (OR) and 95% confidence intervals (95% CI) were computed using unconditional logistic regression to estimate the magnitude of the associations.

**Results:**

Metabolic syndrome was significantly more frequent in females (24.9 vs. 17.4, p < 0.001). In females, the odds favoring metabolic syndrome significantly increased with age and in unfavorable social class as described by occupation, and decreased with education level. In males, metabolic syndrome was significantly more frequent in the 60–69 years age class (OR = 1.82; 95%CI: 1.02–3.26) when compared to those in the 40–49 years age class. Concerning other socioeconomic indicators no significant associations were found.

**Conclusion:**

This study showed that gender influenced the association of socio-economic status indicators with metabolic syndrome. Females in lower social classes, as defined by education and occupational classification, more frequently presented metabolic syndrome, no such association was found in males.

## Background

Metabolic syndrome is characterized by the clustering of conditions that increase cardiovascular and type 2 diabetes risk, including obesity, insulin resistance, dyslipidemia, and high blood pressure. In the recent years, it has internationally evolved into a recognized clinical entity, assuming an epidemic proportion [1-3].

The effective management of the metabolic syndrome requires a better understanding of both molecular mechanisms involved and the population risk profiles. The identification of social and economic characteristics associated with its occurrence is essential for the success of primary preventive measures [4,5].

Studies that addressed social and economic determinants of cardiovascular diseases, consistently showed an increase prevalence of the individual features of metabolic syndrome in the lower socio-economic strata [6-9]. Thus, the prevalence of metabolic syndrome is expected to be influence by social disparities, also providing clues to understand social inequalities in coronary heart disease [10,11]. However, little is known on the association between social and economic factors and metabolic syndrome, considered as a distinct clinical entity, specially addressing gender effects in the social patterning of the disease [12]. It is also recognized that women are particularly sensitive to health inequalities beyond social stratification [13,14], with additional gender effects reflecting such social disadvantage.

Recent reports point to the fact that although metabolic syndrome is only modestly associated with cardiovascular disease risk, the life-course socioeconomic position appears to be an important confounder in that association [15].

This study aimed to assess the association between indicators of past or present social position and the metabolic syndrome in a representative sample of urban non-institutionalised Portuguese adults.

## Methods

As part of a health and nutrition survey a representative sample of the non-institutionalised adult inhabitants Porto, Portugal was recruited. The participants were selected using random digit dialling. When a household was identified, permanent residents were characterized according to age and sex, and one adult was selected by simple random sampling and invited to visit our department for interview and exam. If there was a refusal, replacement was not allowed. The participation rate was 70% [16] and the final sample comprised 2488 subjects aged 18 to 92 years. For this study purpose we evaluated 1962 participants aged 40 years or older, 1207 women (mean age 58.1 ± 11.2 years) and 755 men (mean age 59.0 ± 11.7 years old). The local institutional ethics committee approved the study and all participants provided written informed consent.

Trained interviewers collected information using a structured questionnaire. Data was obtained on social, demographic, personal and family medical history, and behavioural characteristics (physical activity, smoking, alcohol intake, and diet).

Marital status was recorded in four categories: single, divorced, widowed and married, but for analysis purposes, participants were considered as married or not. The number of completed years of formal education was recorded and categorized into four levels: less than five; five to nine; ten to twelve and more than twelve years. Participants currently engaged in a remunerated occupation were classified as active, and the remaining as retired, unemployed or housewives. For those considered active (525 women and 409 men), social and economic status was defined according to their current occupation and the Registrar General five social classes, class I corresponding to the upper social class [17]. Those who were retired, unemployed or housewives, regarding social class were considered as not employed.

Also, information on participant's income and their geographic distribution was collected. Personal income was collected as a categorical variable for approximately 50% of our sample (n = 983). It was however decided not to include this variable in the final analysis because income questions asked in the context of survey research are susceptible to high rates of non-response. Regarding, the geographic distribution of the participants, a preliminary analysis was performed, in which the place of residence was considered, aggregated according to four major town suburbs, by geographical proximity and social characteristics used as an additional surrogate for socio-economic status. However, no difference in the metabolic syndrome prevalence according to the participant place of residence, both in males and in females were found. Additionally, the actual differences in socio-economic characteristics between the classified suburbs could not been accurately establish, so this indicator was omitted from the results.

Anthropometrics were obtained after 12 hours fasting, with the participant in light clothing and barefoot. Body weight was measured to the nearest 0.1 kg using a digital scale, and height was measured to the nearest centimeter, in a standing position using a wall stadiometer. Waist and hip circumference were measured with the subject standing with a flexible and non-distendable tape, avoiding exertion of pressure on the tissues to the nearest centimeter. The waist circumference was measured midway between the lower limit of the rib cage and the iliac crest. The hip circumference was the maximal circumference over the femoral trochanters.

Blood pressure was measured on a single occasion following the American Heart Association recommendations [18], with a standard mercury sphygmomanometer. Two blood pressure readings were taken with the participant resting for 10 minutes, and the mean of the two readings calculated. If the two readings differed more than 5 mm Hg, a third reading was taken and the mean of the two closest readings kept.

Blood was sampled after a 12-hour overnight fast. Glucose, cholesterol (total, high density lipoprotein (HDL), low density lipoprotein (LDL)) and triglycerides were measured using automatic standard enzymatic methods in routine at our Medical School hospital.

Metabolic syndrome was defined according to the Third Report of the National Cholesterol Education Program (NCEP) Expert Panel on Detection, Evaluation, and Treatment of High Blood Cholesterol in Adults (ATPIII) [19], as the presence of at least three of the following characteristics: waist circumference >102 cm in men and >88 cm in women; triglycerides ≥ 150 mg/dl (1.69 mmol/L); HDL cholesterol <40 mg/dl (1.04 mmol/L) in men and <50 mg/dl in women (1.29 mmol/L); blood pressure ≥ 130/85 mm Hg; and fasting glucose ≥ 110 mg/dl (6.1 mmol/L). Participants who reported current antidiabetic or antihypertensive therapy were also considered as having fasting glucose ≥ 110 mg/dl and blood pressure ≥ 130/85 mm Hg respectively.

Metabolic syndrome prevalence was also estimate using the International Diabetes Federation definition (IDF) [20].

### Statistical analysis

Data were analyzed using Stata^®^, release 8. Proportions were compared using the Chi-square test or Fisher's exact test, when appropriated.

To estimate the magnitude of the association between metabolic syndrome and different demographic and socioeconomic factors, crude and adjusted odds ratios (OR) and 95% confidence intervals (95% CI) were computed using unconditional logistic regression.

## Results

The overall prevalence of the metabolic syndrome was 22.0% considering the ATP III criteria and 31.8% when the IDF definition was applied. Metabolic syndrome was significantly more prevalent in females, using both the ATP III (24.9% vs. 17.4%) and the IDF (35.9% vs. 25.6%) definition.

Table 1 presents the prevalence of metabolic syndrome and of its individual features according to the demographic and socio-economic variables evaluated, using ATP III definition. Metabolic syndrome was significantly more common in females (24.9%), in the 60 to 69 years old class (32.3%), in those with less than five years of formal education (27.1%) and in subjects currently unemployed (27.8%).

**Table 1:** Prevalence of metabolic syndrome and its individual features according to demographic and socio-economic characteristics.

	Waist circumference (>102; 88 cm) n (%)	Triglycerides (≥ 150 mg/dL) n (%)	Fasting glucose (≥ 110 mg/dL) n (%)	Blood pressure (≥ 130/85 mm Hg) n (%)	HDL cholesterol (< 40; 50 mg/dL) n (%)	Metabolic syndrome n (%)
Gender						
Female	555 (46.4)	242 (21.5)	148 (13.5)	850 (72.6)	292 (26.2)	300 (24.9)
Male	133 (17.8) *	223 (30.8) *	128 (18.5)	552 (75.9)	131 (18.8) *	131 (17.4) *
Age (years)						
40–49	116 (22.3)	114 (22.7)	36 (7.4)	226 (46.3)	121 (24.3)	60 (11.5)
50–59	192 (34.6)	138 (26.0)	78 (15.0)	404 (72.8)	119 (23.0)	124 (22.0)
60–69	208 (43.5)	135 (29.9)	95 (21.5)	414 (88.5)	107 (24.5)	155 (32.3)
≥ 70	172 (44.0) *	77 (21.3) *	67 (19.3) *	358 (92.7) *	76 (21.3)	92 (23.2) *
Marital Status						
Married	210 (39.7)	111 (22.8)	66 (13.6)	399 (77.0)	146 (30.5)	127 (23.8)
Not married	478 (33.8) *	351 (25.9)	210 (16.1)	1002 (72.8)	276 (20.8) *	304 (21.2)
Education (years)						
≤ 4	436 (47.1)	220 (25.3)	160 (19.0)	742 (81.3)	208 (24.3)	253 (27.1)
5–11	166 (28.6)	144 (26.2)	77 (14.5)	397 (71.1)	120 (22.5)	114 (19.7)
≥ 12	86 (19.7)*	98 (23.3)	38 (9.2) *	260 (61.5) *	93 (22.3)	64 (14.0) *
Occupation						
Active	237 (25.6)	218 (24.6)	97 (11.3)	546 (60.6)	204 (23.5)	145 (15.5)
Retired	307 (41.7)	170 (24.8)	144 (21.5)	641 (88.8)	147 (21.9)	199 (26.8)
Unemployed	28 (41.2)	18 (26.9)	10 (15.9)	50 (75.8)	15 (22.7)	17 (24.6)
Housewives	116 (54.0) *	58 (28.3)	25 (12.5) *	165 (79.3) *	57 (28.1)	70 (32.3) *
Social class						
I	40 (18.6)	51 (24.6)	15 (7.3)	114 (54.3)	40 (19.6)	24 (11.0)
II	22 (12.8)	40 (24.0)	15 (9.5)	99 (57.9)	47 (28.8)	20 (11.4)
III	71 (26.6)	64 (25.6)	32 (13.2)	155 (60.8)	57 (23.6)	45 (16.9)
IV	43 (33.1)	35 (27.1)	20 (16.3)	82 (64.1)	31 (24.4)	29 (22.1)
V	61 (43.3)	28 (20.9)	15 (11.5)	96 (70.1)	29 (21.8)	27 (18.9)
Not employed	451 (44.3) *	246 (25.7)	179 (19.2) *	856 (85.9) *	219 (23.3)	286 (27.8) *

* p < 0.05 – p value for the comparison between classes of the demographic and socio-economic characteristics.

Compared to males, females presented a significantly higher prevalence of central obesity (46.4% vs. 17.8%), low HDL cholesterol levels (26.2% vs. 18.8%) and a lower prevalence of high of triglycerides levels (21.5% vs. 30.8%). All five metabolic syndrome features were present in 3.1% of females and 0.6% of males.

Youngest and more educated participants presented the lowest frequency of individual features of metabolic syndrome, except for low HDL cholesterol and high triglycerides levels.

In females (Table 2), the odds favoring metabolic syndrome significantly increased with age, and decreased with education level and social class, as described by the current occupation. After adjustment for age, body mass index, systolic blood pressure, total physical activity, alcohol consumption and smoking, and compared to more educated women, a higher prevalence of metabolic syndrome was found in women with less than 5 years (OR = 2.28; 95% CI: 1.48–3.51) and with 5 to 11 years of education (OR = 1.49; 95% CI: 0.93–2.36), a significant negative trend being observed. The prevalence of the syndrome was higher in females with no paid job, particularly housewives (OR = 1.77; 95% CI 1.16–2.70). Among active female participants metabolic syndrome was more frequent in those engaged on manual occupations. The prevalence of metabolic syndrome was higher in social class III (OR = 1.85; 95% CI: 0.89–3.85), IV (OR = 2.56; 95% CI: 1.45–5.72), and V (OR = 2.13; 95%CI: 0.97–4.70) when compared to females in the upper social class. A lower risk of metabolic syndrome (OR = 0.70; 95% CI: 0.45–1.07) was found for women with menarche age over 13 years compared with those that menstruated before 12 years of age.

**Table 2:** Prevalence of metabolic syndrome according to socio-economic status, in females.

	Metabolic syndrome n (%)	OR* (95% CI)	OR** (95% CI)
Age (years)			
40–49	38 (11.6)	1	1
50–59	89 (24.5)	2.47 (1.63–3.74)	2.12 (1.39–3.24)
60–69	111 (38.9)	4.85 (3.21–7.34)	3.81 (2.46–5.90)
>69	62 (26.7)	2.77 (1.78–4.33)	2.05 (1.25–3.35)
	<0.001	§	§
Marital status			
Not married	110 (24.3)	1	1
Married	190 (25.2)	1.40 (1.04–1.87)	1.31 (0.97–1.77)
	0.721		
Education (years)			
0–4	202 (31.7)	2.43 (1.61–3.67)	2.28 (1.48–3.52)
5–11	64 (20.4)	1.56 (0.98–2.46)	1.49 (0.93–2.36)
≥ 12	34 (13.3)	1	1
	<0.001	§	§
Occupation			
Active	85 (16.2)	1	1‡
Retired	134 (31.6)	1.64 (1.10–2.44)	1.48 (0.96–2.27)
Housewives	70 (32.6)	1.99 (1.34–2.97)	1.77 (1.16–2.70)
Unemployed	11 (25.6)	1.77 (0.86–3.66)	1.54 (0.73–3.25)
	<0.001		
Social class			
I	13 (10.4)	1	1
II	6 (7.9)	0.74 (0.27–2.05)	0.70 (0.25–1.94)
III	24 (17.9)	1.90 (0.92–3.92)	1.85 (0.89–3.85)
IV	18 (25.6)	2.93 (1.33–6.44)	2.56 (1.45–5.72)
V	24 (19.8)	2.02 (0.98–4.20)	2.13 (0.97–4.70)
Not employed	215 (31.5)	3.01 (1.60–5.64)	2.59 (1.32–4.79)
	<0.001		
Height tertiles (cm)			
135.0–152.4	120 (25.9)	1	1
152.5–157.9	102 (25.0)	1.15 (0.84–1.58)	1.17 (0.84–1.62)
158.0–175.0	78 (23.2)	1.18 (0.83–1.67)	1.20 (0.84–1.72)
	0.676		
Menarche (years)			
≤ 12	134 (25.0)	1	1
13–14	125 (25.7)	1.04 (0.78–1.38)	0.96 (0.71–1.28)
>14	41 (22.4)	0.74 (0.49–1.11)	0.70 (0.45–1.07)
	0.678		

* – OR age adjusted

** – OR adjusted for age, body mass index, systolic blood pressure, total physical activity, alcohol consumption and cigarette smoking.

§ – p for trend <0.05

In males, no social or economic indicators showed a significant association with metabolic syndrome (Table 3), with the exception of height. Males on the second and upper height tertile had an increased risk of metabolic syndrome (OR = 1.67; 95%CI: 1.03–2.68, OR = 1.53; 95%CI: 0.92–2.55, respectively) when compared to those in the lower height tertile.

**Table 3:** Prevalence of metabolic syndrome according to socio-economic status, in males.

	Metabolic syndrome n (%)	OR* (95% CI)	OR** (95% CI)
Age (years)			
40–49	22 (11.2)	1	1
50–59	35 (17.5)	1.68 (0.94–2.98)	1.55 (0.86–2.78)
60–69	44 (22.6)	2.31 (1.32–4.02)	1.82 (1.02–3.26)
>69	30 (18.3)	1.77 (0.98–3.21)	1.38 (0.72–2.64)
	0.026	§	
Marital status			
Not married	18 (21.3)	1	1
Married	113 (16.8)	0.77 (0.43–1.37)	0.86 (0.48–1.57)
	0.330		
Education (years)			
0–4	51 (17.2)	1.08 (0.64–1.81)	1.21 (0.71–2.07)
5–11	50 (18.8)	1.26 (0.76–2.10)	1.30 (0.78–2.18)
≥ 12	20 (14.8)	1	1
	0.585		
Occupation			
Active	60 (14.7)	1	1
Retired	65 (20.4)	1.36 (0.79–2.36)	1.27 (0.72–2.24)
Unemployed	6 (23.1)	1.71 (0.66–4.44)	1.00 (0.33–2.18)
	0.085		
Social class			
I	11 (11.8)	1	1
II	14 (14.1)	1.22 (0.52–2.85)	1.20 (0.51–2.82)
III	21 (15.8)	1.38 (0.63–3.03)	1.62 (0.73–3.60)
IV	11 (17.7)	1.59 (0.64–3.95)	2.22 (0.87–5.68)
V	3 (13.6)	1.16 (0.30–4.59)	1.64 (0.40–6.71)
Not employed	71 (20.5)	1.79 (0.82–3.91)	1.73 (0.79–3.82)
	0.387		
Height tertiles (cm)			
148.2–165.4	40 (14.3)	1	1
165.5–171.3	51 (20.0)	1.61 (1.02–2.56)	1.67 (1.03–2.68)
171.4–189.0	40 (18.7)	1.54 (0.94–2.52)	1.53 (0.92–2.55)
	0.184		

* – OR age adjusted

** – OR adjusted for age, body mass index, systolic blood pressure, total physical activity, alcohol consumption and cigarette smoking.

§ – p for trend <0.05

## Discussion

This study evidenced a gender effect on the association between past and present indicators of social position and the prevalence of metabolic syndrome. Socioeconomic position has been consistently associated with the occurrence of disease. Previous findings on the population distribution of the syndrome's features would make less likely the occurrence of metabolic syndrome in upper socio class strata [4,11,21]. Supporting this hypothesis is the previously described evidence of an inverse graded association between low socioeconomic position and the prevalence of type 2 diabetes and obesity [22-25]. However, the contribution of societal variation to the development of these conditions remains poorly understood.

In Portugal, cardiovascular diseases cause 39% of all deaths and 59% of female deaths [26]. It was also observed a high prevalence of metabolic syndrome, particularly in females [27]. During the second half of the twentieth century, Portugal went through significant social and political transformations that resulted in dramatic changes at the individual level [28], such as obesity prevalence, that doubled during that period [29].

Education is a good indicator of social position in epidemiological studies and often seen as the easier way of measuring present socio-economic status because it precedes other indicators, such as income or occupational based social position, is comparable between women and men, does not usually change in adulthood, and shapes health behaviours through attitudes, values and knowledge. Also, education is considered a good and reliable indicator of childhood socio-economic levels [30]. Our results showed that lower educational levels and occupational categories were significantly associated with the syndrome prevalence in females, these findings being consistent with others from different populations [4,13,31,32]. In a Polish sample, a former communist country going through important social and political transformations, also education was strongly and inversely associated with metabolic syndrome [33]. Likewise, in South Korea another society that experienced considerable changes in its socioeconomic conditions during the past years, socioeconomic inequities in the metabolic syndrome were found in women but not in men [32]. In a Swedish female sample, low education was associated not only with an increased risk of individual risk factors for cardiovascular disease and type 2 diabetes, but also with an increased risk for the metabolic clustering of those factors [31].

However, unlike in other industrialized societies, where a consistent pattern of cardiovascular risk factors distribution is observed according to education, often in a more clear cut manner than for other socio-economic indicators [9,31,33,34], neither education nor occupation were significantly associated with the prevalence of metabolic syndrome in our male participants. This may reflect an unfavourable social and economic female environment, increasing the risk of metabolic syndrome in a gender-specific manner. Also, men in lower socioeconomic strata are more likely to be involved in professional activities more physically demanding, increasing their total energy expenditure, which may also protect them from developing the metabolic syndrome, as previously observed in this population [35].

During the past years, research showed that shorter people were at higher risk of coronary heart disease, stroke and death [36], this increased risk being mainly ascribed to the association of childhood social position with height. Although it is not fully understood, this association seems to be mediated through insulin resistance, a possible consequence of poor childhood nutrition. Also, low childhood socio economic status, is expected to promote adult behavioural risk factors [37,38].

In our sample, no significant association was found between metabolic syndrome and height in females, but in males, shorter stature was associated with a lower frequency of metabolic syndrome. This finding seems rather contradictory to the expected negative correlation between height, social class and cardiovascular disease [39,40]. However, it is consistent with results from a Brazilian sample, where no association was found between total cholesterol, LDL-cholesterol, triglycerides levels and short stature men [41]. Moreover, a recently published review, pointed that shorter southern European or Asian populations presented less cardiovascular disease than taller western Europeans and North Americans, independently of other socio-economic indicators, lifestyles, ethnicity or specific geography [42].

Age at menarche is known to be a sensitive indicator of early life environmental conditions, influenced by factors such as diet, physical activity [43,44], and the magnitude of socio-economic inequalities [45]. Menarche tends to appear earlier in life as the sanitary, nutritional and economic conditions of a society improve [46]. Thus, we would expect an increased risk of metabolic syndrome with increasing menarche age. On the other hand, early menarche age is also associated with an increase in obesity frequency and with insulin resistance [47,48]. In this sample, we observed a trend for a decreased in the adjusted risk of metabolic syndrome with increasing age at menarche. Taken together with the results for height, our data do not favor the hypothesis that these two usual markers of early social conditions increase the risk of metabolic syndrome, as would be expected from previous findings.

### Limitations of the study

Some limitations should however be pointed out. First, the cross-sectional design of our evaluation limits inference regarding causality since data on temporal sequence is lacking.

Secondly, there is no single valid measure of socio-economic status suitable for every population, but financial and time restrains often force researchers to rely on one or two measures to assess socio-economic status. Education is the most widely used measure to control socio-economic status in epidemiological investigation. It is often available for all subjects, has high validity, and it is quite stable after earlier adulthood. This study was not designed to provide a sensitive socio-economic status indicator of metabolic syndrome risk, but education and occupation status were strongly associated with its presence in women, although no such obvious effect was found in men. Different gender related social networks might explain differences in the direction and the magnitude of the observed associations in our sample. Also, as this information was self-reported, some may argued that occupational classification systems differentiate more poorly between women's jobs, and that studies of socioeconomic inequalities in health will underestimate the inequalities more often among women than among men.

Finally, regarding the association between socioeconomic variables, such as social class or occupation and the metabolic syndrome in our male participants, our results may reflect some problems of statistical power, due to the small number of participants in those categories.

## Conclusion

In summary, this study showed that gender influenced the association of socio-economic status indicators and the metabolic syndrome prevalence. Lower social class males and females, defined by occupational classification, presented more frequently metabolic syndrome. Though, occupation is largely dependent on education, only women showed an increasing prevalence of metabolic syndrome with decreasing years of formal education. There was no such education gradient in males.

## Abbreviations

HDL – high density lipoprotein; LDL – low density lipoprotein; NCEP – National Cholesterol Education Program; ATP III – Third Adult Treatment Panel; OR – odds ratio; CI – confidence intervals.

## Competing interests

The author(s) declare that they have no competing interests.

## Authors' contributions

A-CS participated in the design and coordination of the study and performed the statistical analysis and was involved in the drafting of the manuscript. HB was responsible for the study design and coordination. SE and HB were involved the draft critical revising for important intellectual content. All authors read and approved the final manuscript.

## Pre-publication history

The pre-publication history for this paper can be accessed here:


